# CTLA-4 and PD-1 Ligand Gene Expression in Epithelial Thyroid Cancers

**DOI:** 10.1155/2018/1742951

**Published:** 2018-07-05

**Authors:** Chiara Tuccilli, Enke Baldini, Salvatore Sorrenti, Antonio Catania, Alessandro Antonelli, Poupak Fallahi, Francesco Tartaglia, Susi Barollo, Caterina Mian, Andrea Palmieri, Giovanni Carbotta, Stefano Arcieri, Daniele Pironi, Massimo Vergine, Massimo Monti, Salvatore Ulisse

**Affiliations:** ^1^Department of Surgical Sciences, “Sapienza” University of Rome, Rome, Italy; ^2^Department of Clinical and Experimental Medicine, University of Pisa, Pisa, Italy; ^3^Department of Medicine, University of Padua, Padua, Italy

## Abstract

The dysregulation of PD-1 ligands (PD-L1 and PD-L2) and CTLA-4 ligands (CD80 and CD86) represents a tumor strategy to escape the immune surveillance. Here, the expression of *PD-L1*, *PD-L2*, *CD80*, and *CD86* was evaluated at the mRNA level in 94 patients affected by papillary thyroid carcinoma (PTC) and 11 patients affected by anaplastic thyroid carcinoma (ATC). Variations in the mRNAs in PTC patients were then correlated with clinicopathological features. The expression of all genes was deregulated in PTC and ATC tissues compared to normal tissues. In particular, the downregulation of *CD80* was observed above all in ATC. In addition, the increased expression of *CD80* associated with longer disease-free survival in PTC. Higher expression of *PD-L1* associated with the classical histological variant and with the presence of BRAF^V600E^ mutation in PTC. The increased *PD-L2* expression correlated with BRAF^V600E^ mutation and lymph node metastasis, while its lower expression correlated with the follicular PTC variant. The latter was also associated with the *CD80* downregulation, which was also related to the absence of lymph node metastasis. In conclusion, we documented the overall dysregulation of PD-1 and CTLA-4 ligands in PTC and ATC tissues and a possible prognostic value for *CD80* gene expression in PTC.

## 1. Introduction

Based on experimental mouse models and clinical studies, tumor infiltration by cells of the innate and adaptive arms of the immune system is thought to reflect a physiological attempt to eradicate incipient tumor, late-stage cancers, and micrometastases [1–6]. According to the Cancer Immunoediting Hypothesis, the tumor-immune system interaction is divided into three stages: (i) elimination, in which the immune system destroys cancer cells as they arise; (ii) equilibrium, during which the selective pressure of the immune system is thought to promote tumor cell variants capable of evading the immune response; and (iii) escape, where cancer cells are no longer subjected to the immune regulation and tumors develop [7, 8]. The ability of highly immunogenic cancer cells to avoid the immune destruction by disabling components of the immune system is considered a hallmark of cancer [9]. Malignant cells may inactivate cytotoxic T lymphocytes (CTL) and natural killer (NK) cells by secreting immunosuppressive factors, such as TGF-*β*, indoleamine 2,3-dioxygenase, IL-10, and VEGF or through the recruitment of immunosuppressive cells, myeloid-derived suppressor cells (MDSCs), and regulatory T cells (Treg) [10–14].

Malignant cells may also express ligands for the immune checkpoint CTLA-4 (cytotoxic T lymphocyte antigen 4) and PD-1 (programmed cell death 1), present on the surface of activated T lymphocytes, inhibiting their functions [14–19]. Effective activation of T cells requires the engagement of two separate T cell receptors. The antigen-specific T cell receptor (TCR) binds foreign antigenic peptide-MHC complexes, and the CD28 receptor binds to the costimulatory molecules CD80/CD86 (also known as B7.1/B7.2) expressed on the surface of antigen-presenting cells (APC). The simultaneous triggering of these T cell surface receptors with their specific ligands results in T cell activation. In contrast, CTLA-4 (CD152) is a distinct T cell receptor that, upon binding to B7 molecules, elicits an inhibitory intracellular signal [20]. Some studies indicated that B7-CTLA-4 interactions may shield target tumor cells against cytotoxic T lymphocyte- (CTL-) mediated destruction [21].

Differently from B7, the ligands of PD-1, namely, PD-L1 and PD-L2, mediate only suppressive effects on lymphocyte functions by inhibiting the TCR signal. As a consequence, the overexpression of PD-L1/PD-L2 at the surface of malignant cells promotes immune evasion and tumor growth [22]. Over the last few years, antibodies directed against CTLA-4, PD-1, and PD-L1 have shown promising antitumor activities in different cancer types, and the Food and Drug Administration (FDA) approved their use in patients affected by metastatic melanoma and lung cancer [23, 24].

Epithelial thyroid cancer accounts for roughly 1% of all new malignant diseases and 0.4% of deaths related to cancer (http://seer.cancer.gov/statfacts/html/thyro.html). Thyroid tumors comprise the differentiated carcinomas (DTC), that is, the papillary (PTC) and follicular (FTC) carcinomas, and the poorly differentiated (PDTC) and the anaplastic (ATC) thyroid carcinomas [25]. Differently from DTC, usually showing a good prognosis, ATC is unresponsive to all currently available anticancer treatments and leads to death within a few months from diagnosis [26, 27]. The actual staging system for DTC patients (i.e., TNM) incorporates in the same risk group patients showing different disease-specific progression and survival time and fails to predict the risk of cancer recurrences [26, 27]. Thus, the identification of new prognostic molecular markers able to testify thyroid tumor aggressiveness is needed, as well as new therapeutic approaches for the most advanced thyroid cancers [28–31].

A number of studies demonstrated the presence in thyroid cancer tissues of cells belonging to both the innate and adaptive immune systems, including mast cells, NK cells, macrophages, dendritic cells, and B and T cells. Despite that, several experimental findings indicate that the immune response in thyroid cancer is compromised [6, 32–38]. This appears to be related to mechanisms of immune escape put forward by cancer cells, which include the induction of T lymphocyte dysfunction, recruitment of Treg, formation of tolerogenic antigen-presenting cells (APC), and tumor cell expression of immune checkpoint molecules [19, 33].

The analysis of PD-1 ligand expression and their prognostic value is relatively recent in thyroid carcinomas [34, 39–43]. The major part of these studies has been focused on *PD-L1* protein, while only one was conducted on *PD-L1* mRNA [34, 39–43]. Moreover, few studies examined the CTLA-4 ligand (*CD80* and *CD86*) gene expression at the protein level in thyroid cancer cell lines and tissues, including PTC and ATC tissues [44–47].

Considering the importance of the identification of new prognostic molecular markers for aggressive DTC, the necessity of new therapeutic approaches for the most aggressive thyroid cancers [26–31], and the promising results obtained by inhibitors of immune checkpoint molecules in clinical trials [48], the aim of this work was to investigate the expression, at the mRNA level, of CTLA-4 and PD-1 ligands in a case study consisting of 94 PTC and 11 ATC tissues. Variations in the mRNA levels were then correlated with clinicopathological features to evaluate their possible prognostic relevance.

The results suggested a prognostic value for CD80 gene expression in PTC patients and, of note, a role of decreased CD80 expression in ATC progression.

## 2. Materials and Methods

### 2.1. Tissue Samples, Histology, and Patient's Staging

Normal thyroid tissues and matched PTC tissues were obtained from surgical specimens of 94 patients (19 males and 75 females, age range 11–83 yr, median 44 yr) who underwent total thyroidectomy at the Department of Surgical Sciences, “Sapienza” University of Rome (37 patients) or at the Department of Medicine, University of Padua (57 patients) (Supplementary data file (available here)). ATC tissues were collected from surgical specimens of 11 patients (4 males and 7 females, age range 57–79 yr, median 69 yr) who had surgery at the Department of Medicine, University of Padua (6 patients) or at the Department of Clinical and Experimental Medicine of Pisa (5 patients) (Supplementary data file). All the patients gave their informed consent, and the study was approved by the local ethical committee (Protocol number 2615). Tissue samples were collected, quickly frozen in liquid nitrogen, and stored at −80°C until use. Of the 94 PTC patients, 72 exhibited the classical variant, 17 the follicular variant, 3 the tall cells variant, and 2 the oncocytic variant. The histological diagnoses were made independently by two different histopathologists according to the World Health Organization classification [49]. At the time of surgery, lymph node metastases were found in 39 patients. Following TNM staging, 59 patients were at stage I, 1 at stage II, 28 at stage III, and 6 at stage IV. Approximately 40 to 50 days after intervention, all the patients underwent radioiodine treatment followed by thyroid hormone replacement therapy. To ascertain their disease-free condition, 4 to 5 months after intervention, all the patients were controlled by neck ultrasound and serum Tg measurement. Recurrences were diagnosed by determining serum Tg levels either in basal conditions or upon recombinant human TSH stimulation, FNA cytology and/or Tg determination in the FNA washout from lymph nodes, ^131^I whole body scan, and histological analysis of surgically resected lesions. The follow-up included 79 PTC patients (mean 57.1 ± 36.7 months, range 5–141 months), 52 of which were at TNM stage I. During the follow-up, 16 recurrences were recorded (Supplementary data file). Regarding ATC patients, they all died from the disease (survival time range 1–25 months, median 6 months).

### 2.2. Determination of BRAF^V600E^ Mutation

Genomic DNA was extracted from the frozen tissues using the DNeasy Blood and Tissues kit (QIAGEN, Milan, Italy) according to the manufacturer's protocol. The *BRAF* status of exon 15 was assessed by both direct sequencing and mutant allele-specific PCR amplification for the T to A substitution at nucleotide 1799 (V600E), using the procedure previously described [50].

### 2.3. Extraction and Analysis of mRNA

Frozen normal and tumor thyroid tissues were homogenized with the ultraturrax in Isol-RNA Lysis Reagent (FivePrime, San Francisco, CA, USA), and total RNA was extracted. The first cDNA strand was synthesized from 5 *μ*g of RNA with M-MLV reverse transcriptase and anchored oligo[dT]23 primers (Sigma-Aldrich, Milan, Italy). Parallel controls for DNA contamination were carried out omitting the reverse transcriptase. The templates obtained were used for quantitative PCR amplifications of *PD-L1*, *PD-L2*, *CD80*, *CD86*, and three different housekeeping genes (*GAPDH*, *RPL13A*, and *SDHA*), previously validated [30], employing the LightCycler instrument (Roche Diagnostics, Mannheim, Germany), the SYBR Premix Ex Taq II (Tli RNaseH Plus, Takara, Otsu, Shiga, Japan), and specific primers listed in Table 1. Amplicon specificities were checked by automated DNA sequencing (Bio-Fab Research, Rome, Italy), evaluation of melting temperatures, and electrophoresis on 2% agarose gel containing ethidium bromide. Standard curves for all genes were created with fivefold dilutions of a cDNA mix from human thyroid tissue. For the relative quantification of target genes, a normalization factor computed as the geometric media of the 3 reference genes was used, as previously described [28]. The fold changes in gene expression were calculated between each PTC tissue and its normal counterpart, while the ATC samples, for which the normal matched tissues were not available, were compared to a pool of 10 normal thyroid tissues. Data analysis was performed with the Relative Expression Software Tool (REST 2009) using as normalization factor the geometric mean of the abovementioned housekeeping genes, whose expression was proven to be stable among normal, PTC, and ATC tissues in preliminary experiments (data not shown).

### 2.4. Statistical Analysis

The Shapiro-Wilk test was used to evaluate the distribution of data relative to the mRNA level of the different analyzed genes. Then, the nonparametric Mann–Whitney *U* test was used to calculate the statistical significance of differences in the expression levels of the target genes in female versus male patients, in the classical PTC variant versus other variants, in BRAF^V600E^ mutated versus BRAF wild-type (BRAF^WT^) PTC, in metastatic (N1) versus nonmetastatic (N0) PTC, in T_1-2_ versus T_3-4_ tumor sizes, in TNM_I-II_ versus TNM_III-IV_ stages, in the presence or absence of recurrence, and in normal thyroid tissues versus ATC. The nonparametric Fisher's exact test was used to evaluate differences amongst the following groups of patients referring to clinicopathological parameters: (i) BRAF^WT^ versus BRAF^V600E^, (ii) BRAF^WT^ with increased mRNA levels of both *PD-L1* and *PD-L2* versus BRAF^V600E^ with increased mRNA levels of both *PD-L1* and *PD-L2*, (iii) BRAF^WT^ with increased mRNA levels of *PD-L1* and/or *PD-L2* versus BRAF^V600E^ with increased mRNA levels of *PD-L1* and/or *PD-L2*. The correlation of mRNAs with each other and with patient's age was evaluated by Spearman's rho test. The correlation strength was interpreted considering the correlation coefficient value (*r*): 0 < *r* < 0.19, very weak; 0.20 < *r* < 0.39, weak; 0.40 < *r* < 0.59, moderate; 0.60 < *r* < 0.79, strong; 0.80 < *r* < 1.00, very strong [51]. The impact of gene expression on disease-free interval was assessed by the Kaplan-Meier analysis combined with Mantel-Cox log-rank. For the latter, values were classified based on the following criteria: fold change > 1.5 as “increased”; fold change < 0.5 as “decreased”; and 0.5 ≤ fold change ≤ 1.5 as “unvaried.” All statistical analyses were carried out with the SPSS software (IBM, Armonk, NY, USA), and the results were considered significantly different if the pertaining *p* values were lower than 0.05.

## 3. Results

### 3.1. Expression of CTLA-4 and PD-1 Ligands in Papillary (PTC) and Anaplastic (ATC) Thyroid Cancer Tissues

**Figure 1:**
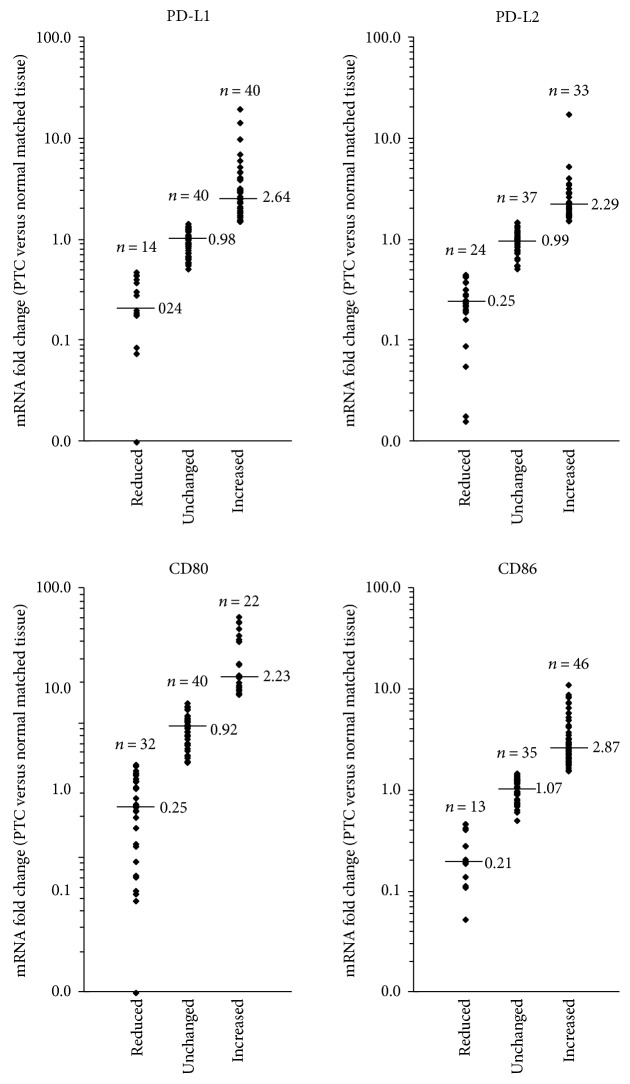
Relative expression levels of *PD-L1*, *PD-L2*, *CD80*, and *CD86* mRNAs in PTC tissues. The fold changes were calculated considering the mRNA values of normal matched thyroid tissues equal to one. Referring to mRNA fold change, three groups are showed (i.e., reduced, unchanged, and increased). For each group, “*n*” indicates the number of cases. The small bars in the graph indicate the median values.

**Figure 2:**
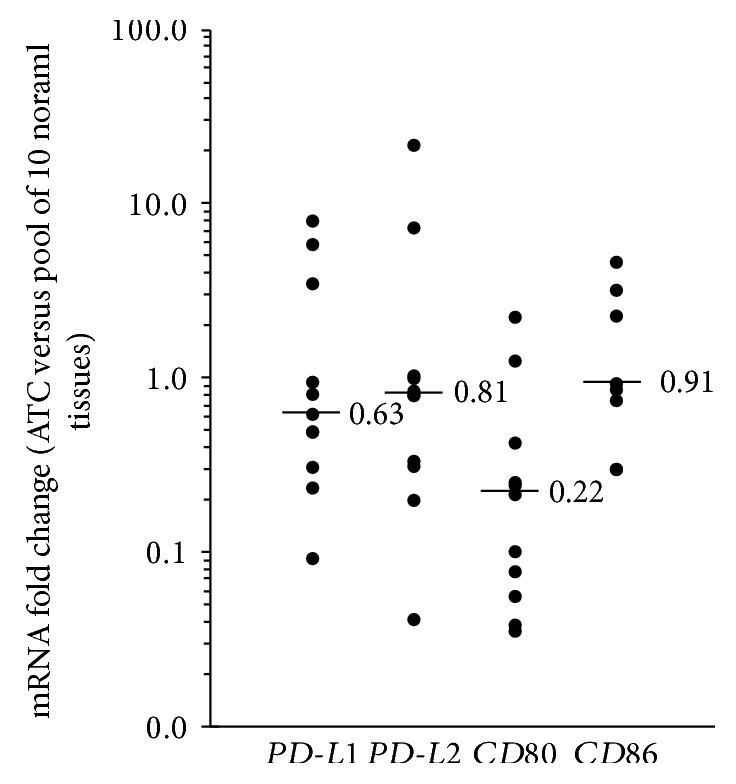
Relative expression levels of the *PD-L1*, *PD-L2*, *CD80*, and *CD86* mRNAs in ATC tissues. The fold changes were calculated considering the mRNA values of a pool of 10 normal thyroid tissues equal to one. The small bars in the graph indicate the median values.

The analysis of the mRNA levels of CTLA-4 and PD-1 ligands in PTC samples, compared to normal matched tissues, revealed that the expression of all transcripts was dysregulated in the majority of cases (Figure 1). Considering a threshold fold change of 0.5 for reductions and of 1.5 for increments, the mRNA levels of *PD-L1* were decreased in 14 (14.9%) and increased in 40 (42.6%) out of 94 PTC tissues analyzed, *PD-L2* mRNA was reduced in 24 (25.5%) cases and increased in 33 (35.1%) cases, *CD80* mRNA level was reduced in 32 (34.0%) and increased in 22 (23.4%) cases, and *CD86* mRNA level was reduced in 13 (13.8%) and increased in 46 (48.9%) cases. The transcript levels were investigated also in 11 ATC tissues, although *CD86* mRNA was analyzed only in 8 ATC samples due to the low amount of the available tissues. Even in ATC, the results showed that the expression of all genes analyzed was deregulated (Figure 2). In particular, the *PD-L1* mRNA was decreased in 3 (27.27%) and increased in 3 (27.27%) out of 11 ATC tissues analyzed, *PD-L2* mRNA was reduced in 4 (36.36%) cases and increased in 2 (18.18%) cases, *CD80* mRNA was reduced in 9 (81.82%) and increased in 1 (9.09%) cases, and *CD86* mRNA was reduced in 2 (25.00%) and increased in 3 (37.50%) cases out of 8 ATC analyzed.

Significant moderate to strong positive correlations were recorded between *PD-L1* and *PD-L2*, *PD-L1* and *CD80*, and *PD-L2* and *CD86* in thyroid cancer tissues (Table 2).

### 3.2. CTLA-4 and PD-1 Ligand Expression in PTC Tissues with and without BRAF^V600E^ Mutation

To assess the effect of BRAF^V600E^ mutation on CTLA-4 and PD-1 ligand expression, we analyzed the mRNA levels of each ligand in 76 PTC tissues for which the BRAF gene status was available. Of these, 38 (50.0%) PTC harbored the BRAF^V600E^ mutation while 38 had the BRAF^WT^. The results of univariate analysis, reported in Table 3, showed that the BRAF^V600E^ mutation associated with increased *PD-L1* and *PD-L2* transcripts in PTC tissues, while no association could be found between BRAF status and CTLA-4 ligands.

In order to assess if the relationship between BRAF^V600E^ mutation and PD-1 ligands had implications on clinicopathological parameters, we performed an analysis comparing the following groups: (i) patients with BRAF^WT^ and increased mRNA levels of both *PD-L1* and *PD-L2* versus patients with BRAF^V600E^ mutation and increased mRNA levels of both *PD-L1* and *PD-L2* and (ii) patients with BRAF^WT^ and increased mRNA levels of at least one PD1 ligand versus patients with BRAF^V600E^ mutation and increased mRNA levels of at least one PD1 ligand. Firstly, we compared BRAF^WT^ versus BRAF^V600E^ patients referring to clinicopathological parameters (Table 4). Significant differences in PTC histology and the TNM stage emerged (Table 4). Nonetheless, by grouping patients based on both BRAF mutational status and augmentation of PD-1 ligand mRNAs, any significant difference was encountered (data not shown).

### 3.3. Association with Clinicopathological Parameters and Prognostic Relevance of CTLA-4 and PD-1 Ligand Expression in PTC Patients

**Figure 3:**
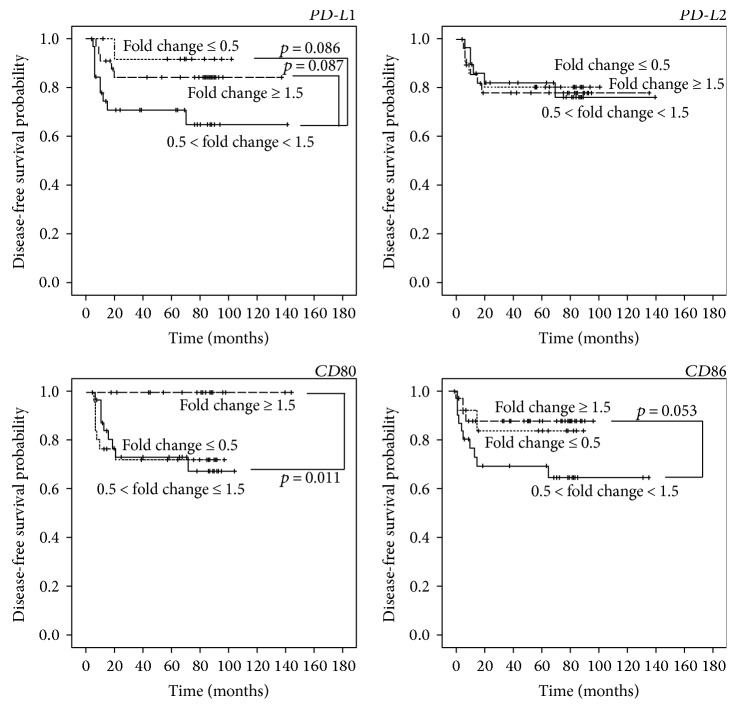
mRNA level of *PD-L1*, *PD-L2*, *CD80*, and *CD86* and disease-free survival (DFS) in PTC patients. Kaplan-Meier analysis combined with Mantel-Cox log-rank statistical test, performed on 79 PTC patients followed up from 5 to 141 months. Expression values of PD-1 and CTLA-4 ligands were classified as increased (fold change > 1.5), decreased (fold change < 0.5), or unchanged (0.5 ≤ fold change ≤ 1.5).

Since an independent association was previously reported between *PD-L1* expression and tissue lymphocytic infiltration [39], we decided to evaluate the fold change of the mRNA level of PD-1 and CTLA-4 ligands in PTC patients with or without thyroiditis. No differences were noticed between the two groups. Likewise, univariate analysis showed no statistically significant association between the mRNA expression of CTLA-4 and PD-1 ligands and gender, age, TNM stage, and recurrences (Table 3). However, a significant association emerged between mRNA levels of *PD-L1*, *PD-L2*, and *CD80* and histology. Specifically, higher *PD-L1* mRNA levels were associated with the classical PTC variant, and lower mRNA levels of *PD-L2* and *CD80* were associated with the follicular PTC variant (Table 3). The statistical analysis also evidenced an association between higher *CD86* mRNA levels and tumor size. Lastly, lymph node metastases were found to associate with increased *PD-L2*, while the reduced *CD80* mRNA levels were related to the absence of lymph node metastases. Kaplan-Meier analysis, performed by dividing the PTC patients in three groups with decreased, unchanged, or increased gene expression, showed that PD-1 ligands did not significantly affect the disease-free survival (DFS). Interestingly, patients with increased *CD80* gene expression had a better DFS (*p* = 0.011) compared to those with either unchanged or decreased *CD80* expression (Figure 3). A similar trend (*p* = 0.053) was observed for *CD86* expression.

## 4. Discussion

The field of cancer immunotherapy promises great results in reducing tumor growth and in achieving complete remission, especially in combination with other therapies. In the past decades, several studies focused on the immune checkpoints mediated by PD-1 and CTLA-4 and their pharmacological inhibition, in order to restrain neoplastic growth [14–19]. The inhibition of one or both the immune checkpoints immediately appeared as a promising therapeutic strategy and, at present, many clinical trials are ongoing for different cancer types, while others were concluded with drug approval by the FDA [23, 24].

The investigation of PD-1 and CTLA-4 ligand expression and their prognostic value is relatively recent in thyroid carcinomas, and only one study examined *PD-L1* expression at the mRNA level [34, 39–43].

Here, we showed that mRNA levels of PD-1 and CTLA-4 ligands in PTC samples were significantly deregulated in the majority of cases, compared to normal matched tissues. Concerning *PD-L1* expression, our data are in agreement with those of previous studies showing an augmented expression in a subset of PTC tissues [34, 39–43]. To our knowledge, no one investigated the expression of *PD-L2* in thyroid carcinomas. In the present study, we found a strong correlation between *PD-L2* and *PD-L1* gene expression, with *PD-L2* upregulated and downregulated, respectively, in 35.1% and 23.4% of PTC cases. Concerning CD80 and CD86, only two previous studies analyzed their expression in PTC by immunohistochemistry, with conflicting results [46, 47]. In our findings, the correlation analysis evidenced a moderate significant covariation between *CD80* and *CD86* mRNA levels in thyroid cancer tissues. In particular, the expression of *CD80* and *CD86* was augmented, respectively, in 23.4% and 48.9% of PTC and diminished in 34% and 13.8% of PTC.

The expression of PD-1 and CTLA-4 ligands was also found deregulated in ATC tissues. Interestingly, *CD80* mRNA was reduced in almost all ATC tissues analyzed. In addition, a lower *CD80* mRNA level was observed to associate with the more aggressive follicular PTC variant. These findings corroborate previous evidence showing reduced expression of CD80 in many cancer types (i.e., melanoma, myeloma, acute myeloid leukemia, bladder, and colon carcinoma) and are in agreement with the observation of its lower expression in poorer differentiated esophageal cancers, compared to well- and moderate-differentiated ones [21, 52, 53]. Thus, it may be tempting to speculate that reduced *CD80* gene expression may confer an advantage to tumor growth. In particular, the lack of CD80 expression has been suggested to be sufficient to evade immune surveillance [51]. In fact, the dysregulated expression of both CD80 and CD86 could negatively affect CTL and Treg functions allowing tumor spread [54, 55]. In addition, transgenic expression of CD80 has been shown to induce tumor regression in xenograft experiments and human clinical trials [56–58]. Considering the different affinity of CD86 and CD80 for CD28 and CTLA-4, respectively, and the constitutive expression of CTLA-4 in Treg cells, cancer cells could induce antigen tolerance towards themselves by modulating Treg cell functions [57]. Such hypotheses appear to be in agreement with our results from Kaplan-Meier analysis, showing a significative association between *CD80* mRNA upregulation with a higher DFS probability in PTC patients. Moreover, although no significative difference was assessed, there was a trend toward a longer DFS when *CD86* was upregulated. In contrast with the hypothesis of protective role of higher *CD80*/*CD86* gene expression, we also found that upregulated expression of CD86 associated with a greater tumor size (pT_3-4_) and that nonmetastatic PTC (pN_0_) had a reduced expression of CD80 compared to metastatic ones (pN_1_). Indeed, these observations were also in contrast with the previously reported results on esophageal cancer, where the lower CD80 protein level was associated with the presence of lymph node metastasis [53]. Although these differences may be due to tumor-specific biology, they also highlight the necessity to corroborate our findings on larger case studies.

Regarding lymph node metastasis, we also observed an association with increased *PD-L2* expression, and conceivably *PD-L1*, at the mRNA level. To our knowledge, no one investigated the relationship between *PD-L2* and thyroid cancers. However, in breast cancer, a significant association between *PD-L2* protein and the lymph node metastasis presence was observed [58].

Concerning the other clinicopathological parameters, in accordance with Shi et al. [42], we observed the higher *PD-L1* mRNA level associated with the classical variant of PTC. The significative difference also interested *PD-L2*, and in our best knowledge, this is the first work that investigates the association of *PD-L2* expression with PTC histology. Differently from Cuhna and colleagues, we did not find other significative associations between PD-1 ligands mRNA and clinicopathological features. In particular, they reported an association between higher tumor stage and the increased *PD-L1* gene expression [39], while we cannot statistically confirm this result, for both *PD-L1* and *PD-L2*, probably because we tested only PTC and a smaller case study.

Differently from the *PD-L1* protein, which other authors demonstrated to be associated with a greater risk of recurrence and with a reduced DFS, our results suggested that the PD-1 ligand mRNA expression cannot be considered as prognostic markers for PTC [40, 42, 46]. The mRNA levels of *PD-L1* and *PD-L2* were almost identical in patients with and without recurrence and did not significantly influence the DFS probability. Further studies on larger cohorts and the parallel analysis of mRNA and protein from the same patients could clarify this aspect.

Lastly, PTC tissues with BRAF^V600E^ expressed higher *PD-L1* levels than BRAF^WT^ tumors. The results appear to be in accordance with those of Angell et al. [34]. Moreover, we also observed a higher *PD-L2* mRNA level in BRAF^V600E^ tumors compared to BRAF^WT^ ones. Our data corroborate the idea that *PD-L1* and *PD-L2* gene expression is a downstream target of BRAF signaling, as previously suggested for PD-L1 in thyroid carcinoma and demonstrated in myeloma [34, 59]. However, our results appear to suggest that the major tumor aggressiveness conferred by BRAF^V600E^ could be independent from the immunosuppressive action of *PD-L1* and *PD-L2*.

## 5. Conclusions

In conclusion, the present work confirmed the overall deregulation of PD-1 and CTLA-4 ligands in PTC and ATC tissues and suggested a possible prognostic value for *CD80* gene expression and a relevant role in ATC progression that could be of interest for the design of novel therapeutic strategies.

## Figures and Tables

**Table 1:** Sequence, exon position, and amplicon size of the primers used in quantification of human PD-1 and CTLA-4 gene expression in thyroid carcinomas.

Gene	Primer sequence	Exon	Amplicon size (bp)
*PD-L1*	5′-TATGCCTTGGTGTAGCACTGAC-3′	5	139
	5′-CCAATGCTGGATTACGTCTC-3′	7	

*PD-L2*	5′-TTTCATAGCCACAGTGATAGCC-3′	5	134
	5′-TCCCAAGACCACAGGTTCAG-3′	7	

*CD80*	5′-CCTGGCTGAAGTGACGTTATC-3′	3	143
	5′-TTTCCAACCAGGAGAGGTGAG-3′	4	

*CD86*	5′-CAACACAATGGAGAGGGAAGAG-3′	6	89
	5′-AAACACGCTGGGCTTCATC-3′	7	

**Table 2:** Correlation among the expression levels of PD-1 and CTLA-4 ligands.

	Correlation coefficient and *p* values			
	PD-L1	PD-L2	CD80	CD86
PD-L1	1.000	0.693	0.539	0.405
	—	<0.001	<0.001	<0.001

PD-L2		1.000	0.644	0.598
		—	<0.001	<0.001

CD80			1.000	0.509
			—	<0.001

CD86				1.000
				—

**Table 3:** Univariate statistical analysis of PD1 and CTLA4 ligand gene expression and PTC patient characteristics and clinicopathological features. For each clinicopathological feature, the number of patients in each group was reported in brackets. For each gene, the median value of mRNA fold change between PTC tissue and its normal counterpart is reported.

	PD-L1	*p* value	PD-L2	*p* value	CD80	*p* value	CD86	*p* value
*Gender*								
Male (*n* = 19)	1.68	0.468	1.48	0.346	0.97	0.210	1.98	0.940
Female (*n* = 75)	1.26		0.99		0.71		1.42	

*Age (y/o)*								
Corr. coeff.	0.131	0.209	0.161	0.122	0.141	0.176	−0.003	0.979

*Hystology*								
Classical variant (*n* = 72)	1.41	**0.002**	1.11	**0.033**	0.89	**0.046**	1.57	0.059
Follicular variant (*n* = 17)	1.02		0.52		0.51		1.27	

*BRAF*								
Wild type (*n* = 38)	1.21	**0.026**	0.86	**0.035**	0.72	0.355	1.21	0.081
V600E (*n* = 38)	1.79		1.41		0.86		1.65	

*pT*								
T_1-2_ (*n* = 39)	1.05	0.060	0.91	0.053	0.63	0.132	1.24	**0.040**
T_3-4_ (*n* = 55)	1.52		1.22		0.92		1.78	

*pN*								
N_0_ (*n* = 55)	1.10	0.062	0.80	**0.002**	0.55	**0.010**	1.47	0.615
N_1_ (*n* = 39)	1.37		1.54		1.02		1.48	

*TNM stage*								
I-II (*n* = 60)	1.12	0.118	0.97	0.113	0.67	0.077	1.43	0.292
III-IV (*n* = 34)	1.54		1.46		0.95		1.88	

*Recurrence*								
No (*n* = 63)	1.28	0.626	1.03	0.903	0.81	0.244	1.39	0.696
Yes (*n* = 16)	1.25		1.03		0.78		1.25	

*Thyroiditis*								
No (*n* = 57)	1.34	0.690	1.07	0.892	0.69	0.129	1.68	0.144
Yes (*n* = 37)	1.10		1.03		0.92		1.35	

**Table 4:** Results from Fisher's exact test to evaluate the association of clinicopathological features and the following groups: BRAF^WT^ (38 patients) and BRAF^V600E^ (38 patients).

	BRAF^WT^	BRAF^V600E^	*p* value
*Gender*			
Male	*n* = 9	*n* = 9	1.000
Female	*n* = 29	*n* = 29	

*Age (y/o)*			
Medium value	41.36	52.17	

*Hystology * ^∗^			
Classical variant	*n* = 27	*n* = 33	**0.046**
Follicular variant	*n* = 9	*n* = 2	

*pT*			
T_1-2_	*n* = 19	*n* = 11	0.100
T_3-4_	*n* = 19	*n* = 27	

*pN*			
N_0_	*n* = 18	*n* = 23	0.357
N_1_	*n* = 20	*n* = 15	

*TNM stage*			
I-II	*n* = 29	*n* = 18	**0.017**
III-IV	*n* = 9	*n* = 20	

*Recurrence * ^∗∗^			
No	*n* = 28	*n* = 28	0.569
Yes	*n* = 9	*n* = 6	

^∗^Other PTC variants were excluded because of their small number. ^∗∗^Information about recurrence was not available for 1 patient in group 1 and 3 patients in group 2.

## Data Availability

The gene expression and patients' clinicopathological data used to support the findings of this study are included within the Supplementary data file.

## Supplementary Materials

Supplementary Materials The supplementary excel file contains all the patients' clinicopathological parameters and gene expression data as described in the Materials and Methods.
