# Supplementary material for: CTLA-4 and PD-1 Ligand Gene Expression in Epithelial Thyroid Cancers
Source: Int J Endocrinol. 2018 Jul 5;2018:1742951. doi: 10.1155/2018/1742951 (PMC6079443; doi:10.1155/2018/1742951)
Supplement: Supplementary Materials — The supplementary excel file contains all the patients' clinicopathological parameters and gene expression data as described in the Materials and Methods. [file 1742951.f1.pdf]

| | mRNA fold change | | | | | |
|---|---|---|---|---|---|---|
| **PATIENT'S ID** | **PD-L1** | **PD-L2** | **CD80** | **CD86** | **SEX** | **AGE AT DIAGNOSIS** |
| **72** | 5.26 | 1.94 | 1.66 | 4.28 | 0 | 35 |
| **74** | 2.40 | 3.61 | 0.92 | 4.98 | 1 | 34 |
| **77** | 1.10 | 0.55 | 0.33 | 0.98 | 1 | 26 |
| **78** | 7.02 | 2.95 | 5.65 | 1.35 | 1 | 49 |
| **80** | 0.18 | 0.28 | 0.81 | 3.04 | 1 | 52 |
| **88** | 1.30 | 0.56 | 1.14 | 0.92 | 1 | 32 |
| **91** | 1.84 | 0.32 | 0.12 | 1.25 | 1 | 33 |
| **98** | 1.95 | 2.97 | 0.51 | 0.64 | 0 | 68 |
| **110** | 2.96 | 3.22 | 1.67 | 3.25 | 1 | 42 |
| **112** | 4.66 | 1.67 | 1.41 | 8.89 | 1 | 54 |
| **115** | 0.52 | 0.24 | 0.24 | 0.51 | 1 | 37 |
| **117** | 0.95 | 0.85 | 0.52 | 0.74 | 1 | 64 |
| **123** | 0.65 | 0.56 | 0.65 | 0.95 | 1 | 67 |
| **131** | 0.59 | 1.19 | 1.09 | 7.34 | 1 | 32 |
| **132** | 1.26 | 1.02 | 0.71 | 7.45 | 1 | 52 |
| **137** | 1.89 | 1.26 | 1.32 | 8.37 | 1 | 43 |
| **140** | 0.92 | 0.87 | 0.63 | 0.80 | 1 | 21 |
| **141** | 1.37 | 1.05 | 1.66 | 1.57 | 1 | 44 |
| **142** | 1.00 | 1.03 | 1.04 | 2.91 | 1 | 43 |
| **144** | 3.16 | 1.83 | 1.33 | 2.72 | 1 | 54 |
| **145** | 1.56 | 1.17 | 0.28 | 2.57 | 1 | 48 |
| **146** | 1.06 | 0.52 | 0.49 | 2.09 | 1 | 24 |
| **149** | 0.87 | 0.80 | 0.37 | 2.99 | 1 | 23 |
| **150** | 0.45 | 0.64 | 0.71 | 0.70 | 1 | 76 |
| **154** | 0.08 | 0.32 | 0.80 | 2.16 | 1 | 56 |
| **156** | 0.44 | 0.24 | 0.05 | 2.28 | 1 | 40 |
| **169** | 0.84 | 0.23 | 0.33 | 1.06 | 1 | 34 |
| **170** | 1.05 | 1.75 | 0.76 | 2.12 | 1 | 64 |
| **263** | 1.52 | 0.43 | 0.95 | 5.89 | 1 | 73 |
| **267** | 1.03 | 0.99 | 1.64 | 0.14 | 1 | 40 |
| **269** | 1.05 | 1.82 | 0.13 | 5.84 | 1 | 35 |
| **270** | 0.48 | 0.44 | 0.44 | 1.20 | 0 | 21 |
| **271** | 1.08 | 0.92 | 0.69 | 6.61 | 1 | 39 |
| **273** | 1.12 | 0.78 | 0.55 | 1.39 | 1 | 51 |
| **276** | 1.26 | 1.56 | 1.02 | 5.33 | 1 | 40 |
| **278** | 0.60 | 0.96 | 0.52 | 1.60 | 1 | 59 |
| **279** | 0.64 | 1.10 | 1.13 | 1.78 | 1 | 46 |
| **p5** | 4.09 | 3.50 | 5.03 | 4.44 | 1 | 64 |
| **p23** | 1.76 | 0.98 | 1.27 | 2.62 | 1 | 38 |
| **p42** | 0.56 | 1.55 | 0.95 | 4.29 | 0 | 52 |
| **p51** | 2.06 | 1.54 | 0.86 | 1.42 | 1 | 59 |
| **p54** | 3.04 | 2.66 | 2.72 | 2.84 | 0 | 65 |
| **p73** | 4.15 | 1.70 | 2.19 | 3.22 | 0 | 47 |
| **p75** | 1.01 | 2.32 | 2.27 | 2.06 | 0 | 34 |
| **p92** | 1.35 | 0.38 | 0.37 | 0.61 | 1 | 54 |
| **p102** | 2.53 | 2.15 | 0.57 | 1.94 | 1 | 25 |
| **p122** | 0.67 | 0.75 | 0.58 | 1.32 | 1 | 60 |

| | | | | | | |
|---|---|---|---|---|---|---|
| **p138** | 1.53 | 0.45 | 0.24 | 0.20 | 1 | 69 |
| **p146** | 0.19 | 0.09 | 0.01 | 0.11 | 1 | 16 |
| **p154** | 1.53 | 0.55 | 0.39 | 0.47 | 1 | 51 |
| **p155** | 0.92 | 2.25 | 4.18 | 2.89 | 0 | 47 |
| **p157** | 0.68 | 1.85 | 0.41 | 0.72 | 0 | 41 |
| **p176** | 1.29 | 1.33 | 1.91 | 1.84 | 1 | 41 |
| **p190** | 2.06 | 1.03 | 0.95 | 1.19 | 0 | 57 |
| **p198** | 2.95 | 0.91 | 0.69 | 1.24 | 1 | 38 |
| **p223** | 0.28 | 0.38 | 0.17 | 1.68 | 1 | 19 |
| **p234** | 3.12 | 1.39 | 1.02 | 1.10 | 1 | 53 |
| **p241** | 2.60 | 2.32 | 0.07 | 1.29 | 1 | 57 |
| **p253** | 0.09 | 0.02 | 0.51 | 0.28 | 1 | 30 |
| **p289** | 2.13 | 4.08 | 1.06 | 1.30 | 1 | 38 |
| **p311** | 0.87 | 1.03 | 0.43 | 0.96 | 1 | 44 |
| **p318** | 1.53 | 1.13 | 0.07 | 1.47 | 1 | 56 |
| **p330** | 1.21 | 0.80 | 1.02 | 0.76 | 1 | 34 |
| **p338** | n.d. | 0.02 | 0.06 | 0.05 | 0 | 42 |
| **p343** | 0.41 | 0.38 | 0.22 | 0.41 | 1 | 32 |
| **p375** | 14.43 | 2.95 | 1.74 | 3.30 | 1 | 60 |
| **p381** | 3.95 | 5.32 | 6.15 | 8.49 | 1 | 61 |
| **p399** | 0.75 | 0.74 | 0.23 | 0.93 | 1 | 53 |
| **P413** | 0.20 | 0.20 | 0.05 | 0.83 | 1 | 52 |
| **p436** | 1.22 | 2.08 | 2.77 | 2.33 | 1 | 43 |
| **p459** | 0.18 | 0.06 | 0.25 | 0.11 | 1 | 59 |
| **p462** | 0.58 | 0.19 | 0.48 | 0.43 | 1 | 69 |
| **p465** | 0.91 | 0.22 | 0.20 | 0.28 | 0 | 50 |
| **p474** | 2.69 | 0.66 | 0.82 | 0.76 | 1 | 40 |
| **p495** | 3.24 | 1.17 | 2.00 | 2.27 | 0 | 38 |
| **p512** | 2.32 | 0.43 | 0.24 | 1.24 | 1 | 73 |
| **p513** | 1.67 | 1.48 | 1.26 | 3.55 | 0 | 34 |
| **p518** | 1.44 | 0.25 | 0.97 | 0.99 | 0 | 11 |
| **p519** | 0.38 | 0.16 | 0.09 | 0.19 | 1 | 45 |
| **p521** | 0.69 | 1.76 | 0.92 | 1.48 | 1 | 15 |
| **p547** | 3.12 | 1.74 | 1.27 | 2.42 | 0 | 62 |
| **p566** | 0.79 | 0.21 | 0.37 | 0.47 | 1 | 36 |
| **p573** | 6.08 | 2.30 | 2.75 | 2.30 | 1 | 59 |
| **p599** | 4.72 | 1.49 | 1.63 | 1.57 | 1 | 36 |
| **p694** | 19.70 | 17.49 | 5.59 | 11.12 | 1 | 43 |
| **p706** | 3.93 | 2.29 | 1.09 | 1.08 | 1 | 44 |
| **p719** | 0.31 | 0.29 | 0.34 | 0.21 | 0 | 55 |
| **CS** | 1.70 | 1.34 | 1.79 | 1.98 | 0 | 48 |
| **BM** | 1.33 | 2.87 | 0.33 | 3.78 | 1 | 40 |
| **C** | 2.34 | 2.38 | 0.87 | 0.92 | 1 | 72 |
| **COL** | 1.26 | 1.07 | 1.85 | 1.17 | 1 | 60 |
| **ML** | 0.74 | 2.92 | 4.04 | 2.49 | 1 | 83 |
| **SL** | 1.58 | 2.03 | 4.48 | 1.27 | 1 | 51 |
| **CA** | 9.92 | 1.22 | 0.42 | 1.90 | 0 | 31 |

| HYSTOLOGY | T | N | M | STAGE | MONTHS OF FU | RECURRENCE |
|---|---|---|---|---|---|---|
| 1 | 3 | 1 | x | 1 | 43 | 0 |
| 1 | 3 | 1 | x | 1 | 10 | 1 |
| 1 | 3 | 1 | x | 1 | 78 | 0 |
| 3 | 3 | 1 | x | 3 | 43 | 0 |
| 2 | 1 | 0 | x | 1 | 102 | 0 |
| 1 | 1 | 1 | x | 1 | 10 | 1 |
| 2 | 3 | 0 | x | 1 | 9 | 1 |
| 2 | 1 | 0 | x | 1 | 85 | 0 |
| 1 | 3 | 0 | x | 1 | 17 | 0 |
| 1 | 3 | 0 | x | 3 | 19 | 0 |
| 1 | 1 | 0 | x | 1 | 14 | 0 |
| 4 | 1 | 0 | x | 1 | 14 | 0 |
| 1 | 1 | 0 | x | 1 | 24 | 0 |
| 1 | 3 | 1 | x | 1 | 10 | 1 |
| 1 | 3 | 0 | x | 3 | 64 | 0 |
| 1 | 3 | 0 | x | 1 | 14 | 0 |
| 1 | 1 | 1 | x | 1 | 69 | 0 |
| 1 | 3 | 1 | x | 1 | 76 | 0 |
| 1 | 3 | 1 | x | 1 | 12 | 1 |
| 1 | 3 | 0 | x | 3 | n.a | n.a. |
| 1 | 1 | 0 | x | 3 | n.a | n.a. |
| 2 | 3 | 0 | x | 1 | n.a | n.a. |
| 1 | 1 | 0 | x | 1 | 38 | 0 |
| 1 | 1 | 0 | x | 1 | 69 | 0 |
| 1 | 3 | 0 | x | 3 | 57 | 0 |
| 2 | 1 | 0 | x | 1 | 56 | 0 |
| 1 | 1 | 0 | x | 1 | n.a | n.a. |
| 1 | 1 | 0 | x | 3 | 39 | 0 |
| 1 | 3 | 0 | x | 3 | n.a | n.a. |
| 2 | 1 | 1 | x | 1 | 21 | 0 |
| 2 | 1 | 0 | x | 1 | n.a | n.a. |
| 1 | 3 | 1 | x | 1 | n.a | n.a. |
| 1 | 3 | 0 | x | 1 | n.a | n.a. |
| 2 | 1 | 0 | x | 1 | 4 | 0 |
| 1 | 1 | 0 | x | 1 | 7 | 0 |
| 2 | 1 | 0 | x | 1 | n.a | n.a. |
| 1 | 3 | 0 | x | 3 | n.a. | n.a. |
| 1 | 3 | 0 | x | 3 | 85 | 0 |
| 1 | 3 | 1 | x | 1 | 90 | 0 |
| 1 | 3 | 1 | x | 4 | 90 | 0 |
| 1 | 4 | 1 | x | 4 | 18 | 1 |
| 1 | 3 | 1 | x | 4 | n.a | n.a. |
| 1 | 1 | 1 | x | 3 | 96 | 0 |
| 2 | 2 | 1 | x | 1 | 94 | 0 |
| 1 | 1 | 0 | x | 1 | 85 | 0 |
| 1 | 3 | 1 | x | 1 | 93 | 0 |
| 1 | 3 | 0 | x | 3 | 11 | 0 |

| | | | | | | | |
|---|---|---|---|---|---|---|---|
| 1 | 3 | 0 | x | 3 | 84 | 0 | |
| 2 | 1 | 0 | x | 1 | 95 | 0 | |
| 1 | 3 | 0 | x | 3 | 83 | 0 | |
| 3 | 3 | 1 | x | 3 | 76 | 0 | |
| 1 | 3 | 1 | x | 1 | 6 | 1 | |
| 2 | 1 | 0 | x | 1 | 87 | 0 | |
| 1 | 1 | 0 | x | 1 | 87 | 0 | |
| 1 | 2 | 1 | x | 1 | 20 | 1 | |
| 1 | 3 | 1 | x | 1 | 89 | 0 | |
| 1 | 3 | 0 | x | 3 | 88 | 0 | |
| 1 | 3 | 1 | x | 4 | 7 | 1 | |
| 2 | 2 | 0 | x | 1 | 66 | 0 | |
| 1 | 1 | 1 | x | 1 | 91 | 0 | |
| 1 | 3 | 1 | x | 1 | 6 | 1 | |
| 1 | 2 | 0 | x | 2 | 89 | 0 | |
| 1 | 3 | 1 | x | 1 | 70 | 1 | |
| 2 | 1 | 0 | x | 1 | 12 | 0 | |
| 2 | 1 | 0 | x | 1 | 70 | 0 | |
| 1 | 1 | 0 | x | 1 | 53 | 0 | |
| 1 | 3 | 0 | x | 3 | 86 | 0 | |
| 1 | 3 | 0 | x | 3 | 85 | 0 | |
| 1 | 3 | 0 | x | 3 | 74 | 0 | |
| 1 | 3 | 1 | x | 1 | 80 | 0 | |
| 1 | 1 | 0 | x | 1 | 90 | 0 | |
| 4 | 3 | 0 | x | 3 | 6 | 1 | |
| 1 | 3 | 0 | x | 3 | 88 | 0 | |
| 1 | 3 | 1 | x | 1 | 88 | 0 | |
| 1 | 1 | 0 | x | 1 | 44 | 0 | |
| 1 | 3 | 0 | x | 3 | 84 | 0 | |
| 1 | 1 | 0 | x | 1 | 85 | 0 | |
| 1 | 4 | 1 | x | 1 | 6 | 1 | |
| 2 | 1 | 0 | x | 1 | 83 | 0 | |
| 1 | 3 | 1 | x | 1 | 15 | 1 | |
| 1 | 3 | 1 | x | 4 | 84 | 0 | |
| 1 | 1 | 0 | x | 1 | 63 | 0 | |
| 3 | 3 | 0 | x | 3 | 66 | 0 | |
| 1 | 1 | 0 | x | 1 | 82 | 0 | |
| 1 | 2 | 1 | x | 1 | 79 | 0 | |
| 1 | 3 | 1 | x | 1 | 76 | 0 | |
| 1 | 3 | 1 | x | 4 | 20 | 1 | |
| 1 | 3 | 1 | x | 3 | n.a | n.a. | |
| 1 | 3 | 1 | 0 | 1 | 6 | 1 | |
| 1 | 3 | 1 | 0 | 3 | n.a | n.a. | |
| 1 | 2 | 1 | 0 | 3 | 141 | 0 | |
| 1 | 3 | 0 | x | 3 | n.a | n.a. | |
| 2 | 3 | 0 | x | 3 | 137 | 0 | |
| 1 | 3 | 1 | x | 1 | n.a | n.a. | |

| BRAF STATUS | THYROIDITIS |
|---|---|
| 1 | 0 |
| 0 | 0 |
| 0 | 1 |
| 1 | 1 |
| 0 | 0 |
| 0 | 1 |
| 0 | 0 |
| 0 | 0 |
| 0 | 1 |
| 1 | 1 |
| n.a. | 1 |
| 0 | 1 |
| 1 | 1 |
| 1 | 0 |
| 1 | 0 |
| 1 | 0 |
| 0 | 1 |
| n.a. | 1 |
| n.a. | 0 |
| n.a. | 0 |
| n.a. | 1 |
| n.a. | 0 |
| 0 | 0 |
| 1 | 1 |
| n.a. | 1 |
| n.a. | 1 |
| n.a. | 0 |
| 1 | 1 |
| n.a. | 0 |
| n.a. | 1 |
| n.a. | 0 |
| n.a. | 0 |
| n.a. | 0 |
| n.a. | 1 |
| n.a. | 0 |
| n.a. | 1 |
| n.a. | 1 |

| | |
|---|---|
| 1 | 0 |
| 0 | 0 |
| 0 | 0 |
| 1 | 0 |
| 1 | 0 |
| 0 | 1 |
| 0 | 1 |
| 0 | 0 |
| 1 | 0 |
| 0 | 1 |

| Legend | | |
|---|---|---|
| **SEX** | Male | 0 |
| | Female | 1 |
| **HYSTOLOGY** | Classical variant | 1 |
| | Folicular variant | 2 |
| | Tall cells variant | 3 |
| | Oncocytic variant | 4 |
| **T** | pT1 | 1 |
| | pT2 | 2 |
| | pT3 | 3 |
| | pT4 | 4 |
| **N** | pN0 | 0 |
| | pN1 | 1 |
| **STAGE** | I | 1 |
| | II | 2 |
| | III | 3 |
| | IV | 4 |
| **RECURRENCE** | No | 0 |
| | Yes | 1 |
| **BRAF STATUS** | BRAF wild type | 0 |
| | BRAF V600E | 1 |
| **THYROIDITIS** | No | 0 |
| | Yes | 1 |
| **n.a.** | Not available | |

| | |
|---|---|
| 1 | 1 |
| 0 | 1 |
| 1 | 0 |
| 1 | 1 |
| 1 | 0 |
| 1 | 1 |
| 1 | 1 |
| 1 | 0 |
| 0 | 0 |
| 0 | 0 |
| 1 | 0 |
| 0 | 0 |
| 0 | 1 |
| 0 | 0 |
| 1 | 0 |
| 0 | 0 |
| 0 | 0 |
| 1 | 0 |
| 1 | 1 |
| 1 | 0 |
| 1 | 0 |
| 1 | 0 |
| 0 | 0 |
| 0 | 0 |
| 0 | 1 |
| 1 | 1 |
| 1 | 1 |
| 1 | 0 |
| 1 | 0 |
| 1 | 0 |
| 0 | 0 |
| 0 | 0 |
| 0 | 1 |
| 0 | 0 |
| 0 | 0 |
| 1 | 1 |
| 0 | 0 |
| 0 | 0 |
| 1 | 1 |
| 0 | 0 |
| 1 | 1 |
| 1 | 0 |
| 1 | 0 |
| 0 | 0 |
| 1 | 0 |
| 0 | 1 |
| 0 | 0 |
